# Grain Sterility in relation to Dry Mass Production and Distribution in Rice (*Oryza sativa* L.)

**DOI:** 10.1155/2014/302179

**Published:** 2014-05-06

**Authors:** Adam B. Puteh, M. Monjurul Alam Mondal, Mohd. Razi Ismail, Mohammad Abdul Latif

**Affiliations:** ^1^Department of Crop Science, Faculty of Agriculture, University Putra Malaysia, 43400 Serdang, Selangor, Malaysia; ^2^Crop Physiology Division, Bangladesh Institute of Nuclear Agriculture, BAU Campus, Mymensingh 2202, Bangladesh; ^3^Institute of Tropical Agriculture, University Putra Malaysia, 43400 Serdang, Selangor, Malaysia; ^4^Bangladesh Rice Research Institute, Gazipur 1701, Bangladesh

## Abstract

The experiment was conducted to investigate potential causes of grain sterility in widely cultivated rice variety in Malaysia, MR219 and its two mutant lines (RM311 and RM109) by examining the source-sink relations. RM311 produced increased dry matter yield both at heading and maturity and also showed higher grain yield with greater proportion of grain sterility than the other two genotypes (RM109 and MR219) resulting in the lowest harvest index (49.68%). In contrast, harvest index was greater in RM109 (53.34%) and MR219 (52.76%) with less grain sterility percentage than MR311 indicating that dry matter partitioning to economic yield was better in RM109 and MR219 than in MR311. Results indicated that dry matter allocation per spikelet from heading to maturity was important for reducing grain sterility in rice. The greater above-ground crop dry matter per spikelet was observed in RM109 and MR219 as compared to high dry matter producing genotype; RM311 implies that poor grain filling may not have resulted from dry matter production or source limitation. These findings suggest that grain sterility or poor grain filling in rice is the result of poor translocation and partitioning of assimilates into grains (sink) rather than of limited biomass production or source limitation.

## 1. Introduction

Rice (*Oryza sativa* L.) is one of the most important food crops in the world. Rice is consumed by more than 50% of the world's population which provides 45–60% of the dietary calories [1]. In the last half century, world rice production has dramatically increased, primarily as the result of genetic improvement. However, rapid population growth and economic development are creating pressures to increase food production.

The yield of rice is an integrated result of various processes including canopy photosynthesis, conversion of assimilates to biomass, and partitioning of assimilates to grains [2]. Further, grain yield can be defined as the product of yield sink capacity and filling efficiency. To further increase yield and to break the yield ceiling, breeding efforts have expanded the yield sink capacity (the maximum size of sink organs to be harvested) mainly by increasing the number of spikelets per panicle. As a result, cultivars with large panicles or extra-heavy panicle types with numerous spikelets per panicle have become available such as the new plant type of the IRRI and hybrid and “super” rice in China [3]. These cultivars, however, frequently do not exhibit their high yield potential due to their poor grain filling, as in a slow grain filling rate and many unfilled grains [1, 4, 5].

The degree and rate of grain filling in rice spikelets differ largely with their positions on a panicle. In general, earlier flowering superior spikelets, usually located on apical primary branches, fill fast and produce larger and heavier grains, while later flowering inferior spikelets, usually located on proximal secondary branches, either are sterile or fill slowly and poorly to produce grains unsuitable for human consumption [6]. The slow grain filling rate and low grain weight of inferior spikelets have often been attributed to a limitation in carbohydrate supply [4]. There are two controversial reports that limited dry matter production caused by low photosynthetic rate and early senescence of leaves during grain filling period or relatively low ratio of source to sink (poor partitioning of assimilates to grain) resulting in poor grain filling of hybrid rice [2, 7]. Other authors reported that hybrid rice showed high dry matter accumulation during grain filling, and their above-ground dry matter per spikelet was greater with higher grain sterility than that of inbred improved varieties, indicating that poor grain filling of hybrid was not a result of source limitation [8]. The poor grain filling might be related to poor partitioning of assimilates to the grain in hybrids.

The indica rice cultivar MR219 (Malaysian rice-219 (MR219) was released as variety) is widely cultivated in Malaysia. The seeds of the popular variety MR219 were treated with sodium azide, the mutagenic agent for creating mutation of the treated seeds. In M_5_ generation, two mutant lines, RM (rice mutant)-311-250-3-6 (RM311) and RM-109-70-2-1 (RM109), were found promising considering yield potential and earliness, respectively. The genotype RM311 can tolerate 10–12 days drought spell at reproductive stage with minor yield reduction, though it is delayed in 7–10 days in maturity than the mother. On the other hand, the genotype RM109 matured 10–12 days earlier than the mother and can escape drought due to shorter growth duration. Moreover, RM311 performed greater yield potential (7.79 t ha^−1^) than the mother MR219 (6.69 t ha^−1^) with more grain sterility percentage, while RM109 performed less yield potential (6.10 t ha^−1^) than the mother with less grain sterility and high harvest index. The objective of this study was to investigate the potential causes of grain sterility (both unfilled and partially filled grain) by examining the source-sink relations in widely cultivated variety MR219 and its two mutant derivative lines. Finding from this study would indicate a possible direction for rice breeding programme aiming at improving the quality of rice crop.

## 2. Materials and Methods

### 2.1. Site Description, Plant Material, and Design

The pot culture experiment was carried out in the glass house of Universiti Putra Malaysia (UPM), during the period from 10 July to 15 November 2012. Geographically, the experimental area is located at 102°12′N latitude and 101°42′E longitudes at the elevation of 31 m above the sea level. Soils were collected from rice growing field, Tanjung Karang, Selangor, Malaysia. The soil was silty clay. The pH value, cation exchange capacity (CEC), and electrical conductivity (EC) of the soil were 5.58, 16.30 meq/100 g soil, and 0.36 dSm^−1^, respectively. The collected soil was well pulverized and dried in the sun. Plant propagules and inert materials were removed from this soil. The dry soil was thoroughly mixed with well rotten cow dung. Triple superphosphate, muriate of potash, and gypsum at the rate of 7.75, 4.92, and 3.75 g pot^−1^ corresponding to 150, 80, and 50 kg ha^−1^, respectively, were mixed with the prepared soil as planting medium in pots. Urea at the rate of 6.75 g pot^−1^corresponding to 125 kg ha^−1^ was applied as three top dressings at 20, 40, and 65 days after planting. The popular rice cultivar MR219 (MR219 denotes Malaysian rice 219) and its NaN_3_-induced mutants RM311 (RM311 denotes rice mutant 311) and RM109 were used as planting materials.

Seeds of the cultivated variety and mutant lines were sown in black perforated plastic polybags of 40 × 45 cm size containing approximately 20 kg of soil obtained from a rice growing area. Several pregerminated seeds were sown in each polybag and plants were thinned to three about two weeks after sowing. The polybags were submerged in water of polyethylene tanks (diameter 100 cm and height 56 cm). A total of eight polybags were put in each polyethylene tank. The polyethylene tanks containing seedlings in polybags were placed in a netted cage and were arranged in a randomized complete block design with eight replications. Standard procedures of rice growing culture were followed throughout the studies. Seedling growth and development were monitored regularly.

### 2.2. Parameters Measured

For measurement of total dry matter production (TDM), plants were sampled at heading and at maturity. One hill per pot was sampled for measurement of TDM. Plant samples were separated into leaf blade, culm, sheath, and panicles. Dry matter of each component was determined after drying at 72°C to constant weight. Leaf area index (LAI) was measured using canopy analyzer (Model: LI-1400, USA) from heading to maturity at ten-day interval. The photosynthesis (Pn) rate in flag leaves was measured using portable photosynthesis system (model: LI-6400XT, USA) at 1000 to 1100 h from heading to maturity stage with seven-day interval. The method for extraction of nonstructural carbohydrate (NSC) was measured following the method of Yang et al. [4]. The sample was dried in an oven and ground into fine powder. In a 15 mL centrifuge tube, 10 mL of 632 g L^−1^ ethanol was added to 100 mg of ground sample and kept in a water bath at 80°C for 30 minutes. The tube was then centrifuged at 5000 rpm for 20 minutes after cooling. The supernatant was collected and the extraction was repeated three times. The alcohol in the supernatant was evaporated on a water bath at 80°C until the alcohol was removed and the volume was reduced to about 3 mL. The sugar extract was then diluted into 25 mL with distilled water. The concentration of sugars in the extract was then analyzed as described by Somogyi [9].

At harvest, morphological parameters, yield components, and grain yield were recorded from two hills of each replication. Percentage of filled spikelet was defined as the number of grains that sank to the bottom of a beaker filled with salt solution with specific gravity of 1.06 as a proportion of total spikelets. Grain plumpness (filled spikelet) percentage was calculated as (1000 fertilized grain weight ÷ 1000 filled grain weight) × 100 [10]. The spikelets which were fully unfilled or partially filled were considered as grain sterility. To evaluate whether the number of sterile spikelets or the number of partially filled grains resulted in low spikelet filling percentage, grain plumpness and the percentages of sterile spikelets and partially filled grains were calculated. Harvest index was calculated by dividing economic yield to biological yield of plant multiplied with 100 and expressed in percentage.

The efficiency of translocation of assimilates and remobilization of stored assimilates in the straw to grains during grain filling period was expressed as transfer ratio of total assimilates. The percentage of remobilized carbon reserve and transfer ratio of total assimilates were calculated as remobilized C reserve = [NSC in stems at heading − NSC residue in stems at maturity] ÷ NSC in stems at heading × 100 [11]. Transfer ratio of total assimilates = [Panicle dry matter at maturity − panicle dry matter at maturity] ÷ [NSC in stems at heading + (plant dry matter at maturity − plant dry matter at heading)].

### 2.3. Statistical Analysis

The collected data were analyzed statistically following the analysis of variance (ANOVA) technique and the mean differences among the treatments were compared by LSD using the statistical computer package program, MSTAT-C.

## 3. Results and Discussion

### 3.1. Grain Yield and Yield Components

The effect of genotypes on grain yield and yield attributes in rice was significant except for the number of tillers hill^−1^ (Table 1). The highest grain yield was recorded in RM311 (29.92 g hill^−1^) due to higher number of tillers hill^−1^, spikelets panicle^−1^, and larger grain size. In contrast, the lowest grain yield was recorded in RM109 (21.14 g hill^−1^) for fewer number of spikelets panicle^−1^. However, filled spikelet (grain plumpness) percentage of RM311 was the lowest (68.9%) which was significantly less than the other two genotypes, RM109 and MR219. The highest filled grain percentage was recorded in MR219 (79.2%) and this genotype also showed the lowest partially filled (12.4%) and unfilled grains (8.4%) followed by RM109 (75.1, 16.1, and 8.8% for filled, partially filled, and unfilled grains, resp.). The results indicate that a greater number of spikelets per panicle did not show maximum efficient yield potential because of their low filled spikelet percentage. Results further revealed that spikelet sterility (unfilled and partially filled spikelets) was positively correlated with number of spikelets panicle^−1^ and grain size (Table 1). Yamamoto et al. [12] reported that the number of spikelets per panicle and per square meter was correlated significantly and negatively with the percentage of ripened grains among 13 rice varieties that supported the present results. But Peng et al. [13] reported that the variation in percentage of filled and partially filled spikelets was not correlated with spikelet number per panicle among 12 tropical japonica rice lines.

### 3.2. Dry Matter Production and Partitioning

**Figure 1:**
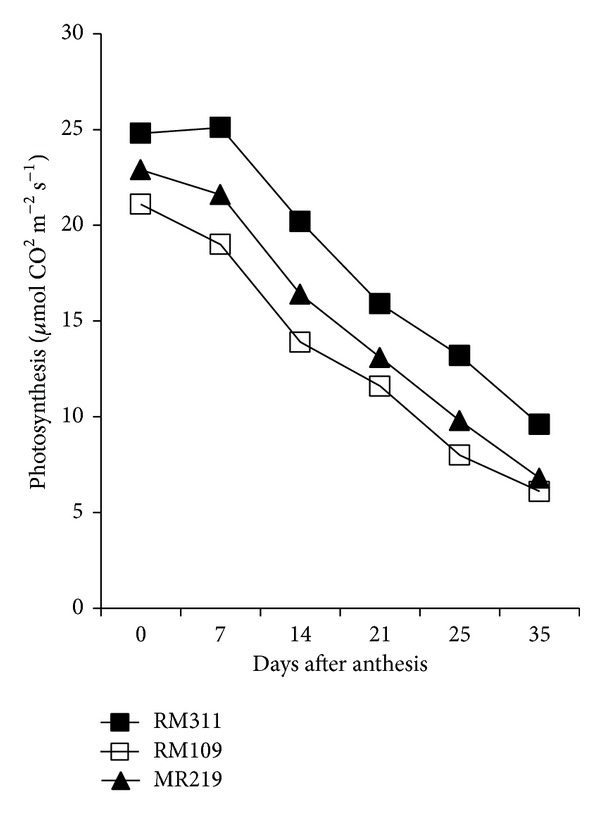
Photosynthesis pattern from heading to maturity.

**Figure 2:**
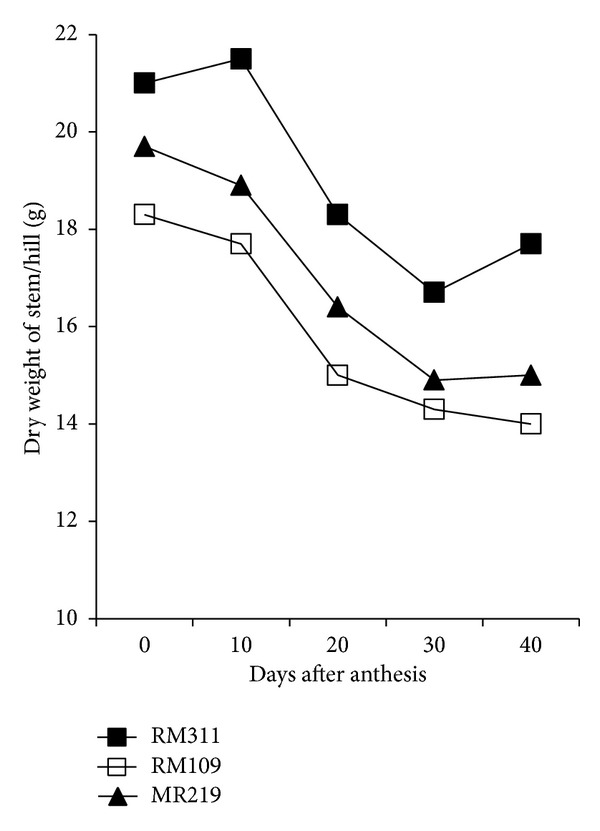
Changes in dry weight of stem during ripening period.

There was a significant variation in above-ground dry matter accumulation and harvest index (HI) in rice genotypes (Table 2). The genotype RM311 produced the highest above-ground dry matter both at heading (40.11 g hill^−1^) and heading to maturity (20.11 g hill^−1^) followed by MR219 (30.37 and 18.47 g hill^−1^ for heading and heading to maturity, resp.). The greater biomass production both at heading and heading to maturity of RM311 associated with higher leaf area index (LAI) and photosynthesis (Pn) rate (Figures 1 and 2) during the grain filling period. In contrast, the lowest above-ground dry matter was recorded in RM109 for its lower LAI and Pn rate. Although RM311 produced the highest total dry mass (TDM) but had the lowest HI (49.68%) indicating that the dry matter partitioning to economic yield was inefficient in RM311, HI was greater in RM109 and MR219 than in MR311 indicating that dry matter partitioning to economic yield was better in RM109 and MR219 than in RM311. Based on total spikelets production, HI would be the highest in RM311 (68.32%) followed by RM109 (64.74%) and MR219 (61.55%). In contrast, MR219 showed the lowest expected HI with higher actual HI indicating that assimilate was more efficiently partitioned in MR219 than the other two genotypes. However, results showed that maximum dry matter production at heading had positive correlation with grain sterility. For example, RM311 produced the highest TDM (40.11 g hill^−1^) at heading with the highest unfilled grains (12.1%), while RM109 produced the lowest TDM at heading (25.12 g hill^−1^) with less unfilled grains (8.8%). Results further revealed that the proportion of dry matter production from heading to maturity was greater in MR109 (37.8%) and MR219 (38.8%) than in RM311 (33.4%) suggesting that the increase in dry matter accumulation after heading would be greatly beneficial to enhance grain filling process because 80–100% of the yield comes from assimilates which are produced during grain filling period [14]. This result is consistent with Yang et al. [4] who reported that higher TDM production during grain filling was beneficial for grain filling in rice.

Considering the dry matter allocation per spikelet, results showed that dry matter accumulation from heading to maturity was higher in MR219 and RM109 than in RM311 (Table 3), though the later genotype produced greater TDM than the earlier two genotypes (Table 2). The highest dry matter allocation per spikelet was recorded in MR219 (14.22 mg spikelet^−1^) and also showed the lowest grain sterility (20.8%, partially filled and unfilled grains). The greater above-ground crop dry matter per spikelet was found in RM109 and RM219 with lower LAI as compared to high TDM producing genotype (RM311) which implies that poor grain filling may not have resulted from dry matter production or source limitation. This result indicates that grain sterility is mainly due to insufficient assimilate supply to grain. The percentage of remobilized carbon (C) reserve and transfer ratio of total assimilate (TRA) to grains during grain filling period were lower in RM311 (30.9% and 0.64, resp., for remobilized C and TRA) than the other two genotypes, RM109 (48.2% and 0.74 for remobilized C and TRA, resp.) and MR219 (50.5% and 0.82 for remobilized C and TRA, resp.). The low remobilization of assimilates of RM311 resulted in a large amount of nonstructural carbohydrate (NSC) left in stems at maturity (106.6 mg g^−1^ dw) causing greater grain sterility (partially filled and unfilled grains), thereby poor harvest index. These results indicate that grain sterility is mainly due to low translocation rate from source to sink since little C reserve was remobilized and much of the NSC remained in the stems at maturity.

### 3.3. Photosynthesis, Stem Dry Weight, and Leaf Area Index

**Figure 3:**
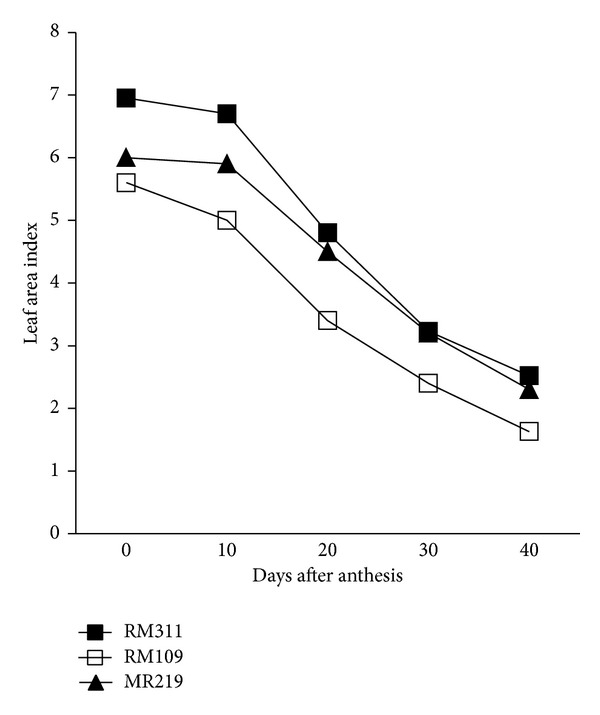
Variation in leaf area index from heading to maturity of three rice genotypes.

The pattern of photosynthesis (Pn) rate and dry weight of stem during the grain growth period were different among the genotypes (Figures 1 and 2). Pn rate decreased with age from heading to maturity in all the genotypes but decreasing of Pn rate with time was less in RM311 than the other two genotypes, RM109 and MR219 (Figure 1). The lowest Pn was recorded in RM109 followed by MR219. A similar result was also observed in case of stem dry weight (Figure 2). Results indicated that production of dry matter had positive relationship with Pn rate. The genotypes which showed higher Pn rate also produced high dry matter (DM). Similarly, leaf area index (LAI) decreased with age from heading to maturity in all the genotypes due to leaf senescence. The highest LAI from heading to maturity was recorded in RM311 followed by MR219 and RM109 (Figure 3). Considering LAI decrease in percentage over age, the result showed that, during early to middle grain growth stages, the decrease in LAI was the lowest in MR219 (not shown in tables or figures but calculated from Figure 3). This indicates that green leaf area duration was higher in MR219 at reproductive stage than the other two genotypes, RM109 and RM311. This longer leaf area duration thus produced more assimilates and supply assimilates sufficient to grains and resulted in the highest grain plumpness/lowest grain sterility in MR219.

Based on source-sink relationships, rice varieties can be categorized as sink-limiting, source-limiting, or intermediate types [15]. Most indica inbred/hybrid rice belongs to the source-limiting type, with a high ratio of spikelet number to leaf area at heading and low filled grain percentage. In this study, we found that RM311 had both greater number of spikelets per panicle and greater biomass production but had the highest grain sterility due to the little C reserve being remobilized and much of NSC remaining in the stems at maturity. This result strongly suggests that poor grain filling of RM311 was a result of poor translocation and partitioning of assimilates into grains rather than limited biomass production or source limitation. Therefore, it may be concluded that understanding the mechanism of poor translocation and partitioning of assimilates into grains may help to improve the proportion of filled grain and thereby increase yield. MR219, the popular cultivar in Malaysia, had greater assimilate remobilization pattern during grain growth and development resulting in the highest filled grain percentage with low grain sterility.

## 4. Conclusion

Efficient and higher dry matter allocation per spikelet is more important than increasing the total dry matter production to reduce grain sterility in rice. Grain sterility is mainly due to low translocation rate from source to sink rather than limited biomass production or source limitation. Therefore, it is possible to improve grain filling of indica rice by selecting suitable parents.

## Figures and Tables

**Table 1:** Grain yield and yield components of rice genotypes.

Genotypes	Grain yield (g hill^−1^)	Number of tillers hill^−1^	Number of spikelets panicle^−1^	Number of filled spikelets panicle^−1^	Grain plumpness (%)	Partially filled grains (%)	Unfilled grains (%)	Grain weight (mg grain^−1^)
RM311	29.92a	9.80^ns ^	202^a^	139.2^a^	68.9^b^	19.0^a^	12.1^a^	25.67^a^
RM109	21.14c	9.51	136^c^	102.1^c^	75.1^a^	16.1^b^	8.8^b^	24.27^ab^
MR219	25.63b	9.57	153^b^	121.2^b^	79.2^a^	12.4^c^	8.4^b^	23.86^b^

The same letter in a column does not differ at *P* ≤ 0.05 by LSD.

**Table 2:** Above-ground dry matter accumulation (g hill^−1^) and harvest index of rice genotypes.

Genotypes	At heading	Heading to maturity	Entire growing period	Harvest index (%)	Expected harvest index (%)*	Gap (%)
RM311	40.11^a^	20.11 a (*33.4%* ^b^)	60.22^a^	49.68^b^	68.32^a^	18.64^a^
RM109	25.12^c^	14.52 b (*37.8%* ^a^)	39.64^c^	53.34^a^	64.74^b^	11.4^b^
MR219	30.37^b^	18.47 a (*38.8%* ^a^)	48.84^b^	52.76^a^	61.55^b^	8.79^b^

The same letter in a column does differ at *P* ≤ 0.05 by LSD; *calculated based on total spikelet number; figures in parenthesis indicate % of dry matter contribution.

**Table 3:** Above-ground crop dry matter per spikelet and remobilization of stored assimilates from straw to panicle.

Genotypes	At heading (mg spikelet^−1^)	Heading to maturity (mg spikelet^−1^)	Entire growing period (mg spikelet^−1^)	Remobilized carbon reserve^†^ (%)	TRA^‡^	NSC residue (mg g^−1^ dw)	Partially filled and unfilled grains (%)
RM311	22.04^ns ^	11.32^b^	33.36^b^	30.9^b^	0.64^b^	106.6^a^	31.1^a^
RM109	21.42	13.07^a^	34.49^b^	48.2^a^	0.74^ab^	76.4^b^	24.9^b^
MR219	22.83	14.22^a^	37.05^a^	50.5^a^	0.82^a^	63.5^c^	20.8^b^

The same letter in a column does not differ at *P* ≤ 0.05 by LSD; ^†^(nonstructural carbohydrate (NSC) in stem at heading − NSC residue in stem at maturity) ÷ NSC in stem at heading × 100; ^‡^transfer ratio of total assimilates = (panicle dry matter at maturity − panicle dry matter at heading)  ÷  [NSC in stem at heading + (plant dry matter at maturity − plant dry matter at heading)]; residue = the amount of NSC remaining in stem at maturity.
